# Serum albumin levels and risk of atrial fibrillation: a Mendelian randomization study

**DOI:** 10.3389/fcvm.2024.1385223

**Published:** 2024-04-09

**Authors:** Bohang Chen, Chuqiao Wang, Wenjie Li

**Affiliations:** ^1^ The First Clinical Medical College, Liaoning University of Traditional Chinese Medicine, Shenyang, Liaoning, China; ^2^ Department of Cardiovascular Medicine, Affiliated Hospital of Liaoning University of Traditional Chinese Medicine, Shenyang, Liaoning, China

**Keywords:** serum albumin, atrial fibrillation, causal relationship, Mendelian randomization study, mediating effect, serum metabolites

## Abstract

**Objective:**

Although several observational studies have linked serum albumin to cardiovascular disease and considered it as an important biomarker, little is known about whether increasing or maintaining serum albumin levels can effectively improve the prognosis of patients with atrial fibrillation. Therefore, this study aims to further explore the causal relationship between serum albumin and atrial fibrillation and its potential mechanism.

**Method:**

Using data from large-scale genome-wide association studies, we conducted a two-sample Mendelian randomization (MR) analysis and a mediation MR analysis, using serum albumin as the exposure variable and atrial fibrillation as the outcome variable. We included 486 serum metabolites as potential mediating factors. To increase the robustness of the analysis, we applied five statistical methods, including inverse variance weighted, weighted median, MR-Egger, simple mode, and weighted mode. Validate the MR results using Bayesian weighted Mendelian randomization method.

**Result:**

The results of the MR analysis indicate a significant inverse association between genetically predicted serum albumin concentration (g/L) and the risk of atrial fibrillation (Beta = −0.172, OR = 0.842, 95% CI: 0.753–0.941, *p* = 0.002). Further mediation MR analysis revealed that serum albumin may mediate the causal relationship with atrial fibrillation by affecting two serum metabolites, docosatrienoate and oleate/vaccenate, and the mediating effect was significant. In addition, all our instrumental variables showed no heterogeneity and level-multiplicity in the MR analysis. To verify the stability of the results, we also conducted a sensitivity analysis using the leave-one-out method, and the results further confirmed that our findings were robust and reliable. Finally, we conducted a validation using the Bayesian weighted Mendelian randomization method, which demonstrated the reliability of our causal inference results.

**Conclusion:**

This study strongly demonstrates the causal relationship between serum albumin and reduced risk of atrial fibrillation through genetic methods, and reveals the key mediating role of two serum metabolites in this relationship. These findings not only provide a new perspective for our understanding of the role of serum albumin in atrial fibrillation, but also provide new ideas for the prevention and treatment strategies of atrial fibrillation.

## 1 Introduction

Cardiovascular disease has become the leading cause of death worldwide. Epidemiological factors indicate that low serum albumin levels are independently associated with a variety of cardiovascular diseases, including coronary artery disease, heart failure, and atrial fibrillation, and have been established as effective and independent prognostic indicators for patients with cardiovascular disease (1). Through multiple long-term and large-scale cohort studies, a significant trend has emerged: a decrease in serum albumin levels is significantly correlated with a relative increase in the incidence of atrial fibrillation (2, 3). A comprehensive meta-analysis involving 9 trials and 32,130 subjects further strengthened this view. The analysis showed that compared to patients with low serum albumin levels, patients with high serum albumin levels had a significantly reduced risk of developing atrial fibrillation (OR: 0.62, 95% CI: 0.44–0.89, *P* = 0.009). Specifically, every 10 g/L increase in serum albumin level was associated with a 36% reduction in the relative risk (95% CI: 0.51–0.81, *I*^2^ = 87%, *P* < 0.001) (4). However, although observational studies have shown consistency in revealing the negative correlation between serum albumin levels and atrial fibrillation, there is still uncertainty regarding whether preventing and correcting low serum albumin has actual benefits for the prognosis or patients at risk of atrial fibrillation. In other words, the potential causal relationship between the two has not been clearly confirmed, which remains a focus of current controversy. In view of this, we urgently need to conduct more in-depth research to clarify the causal relationship between serum albumin levels and atrial fibrillation and its underlying mechanisms. This will provide important theoretical basis and practical guidance for future cardiovascular disease prevention, treatment, and rehabilitation. At the same time, it will also help us to more fully understand the important role of serum albumin in cardiovascular disease, laying a solid foundation for the development of new treatment strategies and methods.

Mendelian randomization (MR) is an effective method for evaluating the causal relationship between observable variable exposures or risk factors and clinical outcomes (5). In particular, when randomized controlled trials are limited in verifying causal relationships, and observational studies exhibit biased associations due to potential confounding variables or reverse causality, this genetic analysis method highlights its importance (6). In this study, we systematically collected published data and employed a two-sample Mendelian randomization analysis method to identify the causal relationship between serum albumin level and atrial fibrillation through rigorous data processing and analysis procedures, aiming to reveal the true and unbiased association between the two.

## 2 Materials and methods

### 2.1 Source of data

We collected the genome-wide association study (GWAS) dataset related to serum albumin level and atrial fibrillation by visiting the website (https://gwas.mrcieu.ac.uk). Among them, the GWAS dataset for serum albumin levels (*N* = 400,938) was derived from a study utilizing exon-wide imputation, association, and fine-mapping (7). This study accurately inferred exon-wide variations by leveraging extensive haplotype sharing between 49,960 UK Biobank participants with exome sequencing and the rest of the cohort. The aim was to determine the causal effects of rare coding variants on 54 quantitative traits. The GWAS dataset for atrial fibrillation was obtained from a study that aggregated a large-scale individual data from the Atrial Fibrillation Genetics (AFGen) Consortium (Ncase = 15,979, Ncontrol = 102,776) (8). Through a comprehensive and meticulous meta-analysis of 33 studies, including GWAS, Exome-Wide Association Studies (ExWAS), and Rare Variant Association Studies (RVAS), a high-quality GWAS dataset tailored for atrial fibrillation was constructed. This dataset not only encompasses common variants but also delves into the impact of rare variants on atrial fibrillation, further elucidating the genetic basis of the condition.

In addition, we also obtained a serum metabolite dataset from a large-scale GWAS study (ID range: GCST90199621-GCST90201020). This study comprehensively examined two European population-based surveys involving a total of 7,824 participants. With the help of advanced technology and strict quality control processes, the study explored the genetic basis of more than 400 blood metabolites, and ultimately identified 486 metabolites at the genetic level for our subsequent analysis (9). Among these metabolites, 309 are known and 177 are unknown. In order to more systematically understand these known metabolites, we summarized them into eight major metabolic pathways, each involving different biochemical processes (10).

In this analysis, no sample overlap was found among the three GWAS datasets we used. The above datasets provide us with rich genetic information and biochemical background, which helps us to explore the association between serum albumin and atrial fibrillation and its potential genetic and metabolic mechanisms in depth.

### 2.2 Research method

In this study, we used R4.2.2 statistical software combined with professional R packages such as TwoSampleMR and MR-PRESSO to conduct systematic MR analysis to further explore the possible causal relationship between serum albumin and atrial fibrillation. To ensure the accuracy and robustness of the research results, we comprehensively applied five advanced statistical methods, including inverse variance weighted, weighted median, MR-Egger, simple mode, and weighted mode methods. Among these methods, the inverse variance weighted (IVW) method became the main method for our MR analysis due to its efficient and stable characteristics.

Bayesian weighted Mendelian randomization is a statistical method that combines Bayesian inference with MR (11). It utilizes MR to eliminate the influence of confounding factors and employs Bayesian inference to integrate prior information with observed data, obtaining a posterior distribution for more accurate estimation of causal relationships. Specifically, this approach can weight the effect sizes of exposure factors based on prior information and sample data, ensuring that exposure factors with larger effects have a greater influence in the analysis. This enhances the comprehensiveness and reliability of our analytical results.

### 2.3 Instrumental variable assumption

In the process of using genetic variation to assess causal effects, selecting SNPs as instrumental variables is crucial. We must strictly follow three basic assumptions to ensure the accuracy and reliability of the study. First, the selected SNPs must be significantly correlated with the exposure factor; second, the association between the SNP and the outcome must be independent of any confounding factors; finally, the SNP can only be linked to the clinical outcome through the exposure factor (12).

To avoid bias due to linkage disequilibrium, we set strict screening criteria. Specifically, SNPs significantly associated with exposure factors, in the presence of linkage disequilibrium, need to satisfy an r^2^ value of less than 0.001 and a genetic distance of 10,000 kb. To ensure the robustness of the selected instrumental variables, we also conducted further testing using the F statistic. Only when the F value is greater than 10 can we be confident that there is no weak instrumental variable bias (13).

When screening SNPs associated with serum metabolites, we adopted a relatively loose statistical threshold (*P* < 1e-05). This strategy aims to ensure that as many exposure variants as possible can be captured even with a small number of significant SNPs. However, we did not lower the quality requirements for instrumental variables. Instead, we still adhere to strict linkage disequilibrium conditions (*r*^2^ value less than 0.01, genetic distance not exceeding 500 kb) and the F > 10 criterion for eliminating weak instrumental variables.

After a series of rigorous screening processes, we successfully extracted instrumental variables that met all conditions and included them in the scope of the study. At the same time, we also recorded detailed information about each SNP's effect allele (EA), allele effect value (β), standard error (SE), and *P* value (14, 15).

### 2.4 Statistical method

We conducted detailed data analysis using statistical software version R4.2.2 and its dedicated R packages (TwoSampleMR and MR-PRESSO). To ensure the accuracy and consistency of the data, we carefully summarized the statistical information of the exposure factor and clinical outcome datasets, and checked the allelic correspondence of each SNP on the effect of exposure factors and clinical outcomes one by one. The IVW method, as the core analysis method, provides a comprehensive weighted assessment of potential causal effects by integrating the MR effect estimates of each SNP (16). This method is most reliable when there is no level heterogeneity in instrumental variables (17). At the same time, the weighted mode method can robustly estimate causal effects even when up to 50% of the information comes from genetic variants with invalid instrumental variables (18).

To test whether there is horizontal multilevel effect in instrumental variables, we used the MR-Egger regression method, which effectively estimates the impact of horizontal multilevel effect through the intercept term (19). To further detect and correct horizontal multilevel effect, we also used the MR-PRESSO test to achieve this goal by removing outliers (20). In assessing heterogeneity of instrumental variables, we used Cochran's *Q*-test for quantitative analysis. When the *P* value is less than 0.05, we determined that there is significant heterogeneity, and at this time, we will choose the IVW random effect model to estimate causal effects to ensure the robustness of the results. Throughout the analysis process, we set *P* < 0.05 as the threshold to determine whether the results have statistical significance.

## 3 MR result

### 3.1 Causal relationship between serum albumin level and atrial fibrillation

We set serum albumin levels as the exposure factor, atrial fibrillation as the study outcome, and set strict statistical significance thresholds (*P* < 5e-08). After screening, we have preliminarily identified 208 SNPs associated with serum albumin levels. However, based on our hypothesis of linkage disequilibrium, we further excluded 7 SNPs. These 7 SNPs are rs10743939, rs11871801, rs17151639, rs2696671, rs3744274, rs7797854 and rs930734. Next, to avoid the issue of weak instrument variable bias, we set a screening condition with an F-test value greater than 10. After this round of screening, we further eliminated 46 SNPs. These 36 SNPs are rs12209602, rs12453576, rs12516449, rs1675382, rs1782455, rs1809423, rs1973878, rs2228213, rs2301029, rs2736231, rs27744, rs28925904, rs3087243, rs3213868, rs33994242, rs3756772, rs4730221, rs55910200, rs57826934, rs61735533, rs62622830, rs648514, rs6684353, rs67611724, rs6873349, rs7031621, rs704017, rs7153110, rs725660, rs74010640, rs74397112, rs7443182, rs75009793, rs755731, rs76701589, rs7726159, rs8045125, rs8187658, rs843925, rs848486, rs869337, rs870526, rs921071, rs921835, rs9309325 and rs9772460. To ensure the purity of instrumental variables, that is, the association between SNP and outcome is not affected by confounding factors, we carefully searched the PhenoScanner database. The results showed that 36 SNPs were associated with thyroid disease, diabetes, hypertension, coronary artery disease and other potential confounding factors. These confounding factors may interfere with our research results, so we have decided to exclude these SNPs from the analysis. These 36 SNPs are rs4409785, rs1133400, rs6897932, rs2586886, rs460879, rs12243326, rs6486122, rs6861681, rs7586970, rs4804416, rs1076540, rs1378942, rs1229984, rs10468017, rs10828249, rs10517086, rs2923089, rs58148580, rs2972143, rs6935921, rs459193, rs646776, rs9088, rs4540292, rs2000999, rs2229357, rs653178, rs731839, rs1128249, rs13107325, rs17036326, rs1122326, rs6831256, rs9987289, rs1260326 and rs28929474. Before conducting the MR analysis, we performed quality control on the 119 SNPs obtained through the aforementioned screening process, filtering out those with ambiguous base calls. Ambiguous base calls refer to situations where the specific base at a particular position cannot be unambiguously determined, which may result from sequencing errors, sample contamination, or other factors. The presence of ambiguous base calls can introduce bias into our analysis, affecting the accurate inference of genotypes and subsequently impacting the estimation of causal effects as well as the statistical power of the analysis. If ambiguous base calls are present, we will remove them to ensure the accuracy and reliability of our analysis results. We didn't find any ambiguous base calls, so no SNPs were removed in this step. Finally, we identified 119 robust SNPs as instrumental variables for subsequent MR analysis. Through this series of screenings for SNPs, we have ensured that our analysis has adequate statistical power.Detailed information of SNP is shown in Supplementary Table S1.

After a series of rigorous MR analyses, we consistently found a significant causal relationship between the increase in serum albumin levels and the reduction in the risk of atrial fibrillation. This conclusion was supported by five different MR analysis methods, all of which had an odds ratio (OR) less than 1, strongly supporting our research hypothesis. It is particularly noteworthy that we used the IVW method as the primary analysis method. The IVW method has high statistical power and can more accurately reveal potential causal relationships. The analysis results showed that there was a significant association between the increase in serum albumin levels and the reduction in the risk of atrial fibrillation (IVW: Beta = −0.172, OR = 0.842, 95% CI: 0.753–0.941, *P* = 0.002).

**Figure 1:**
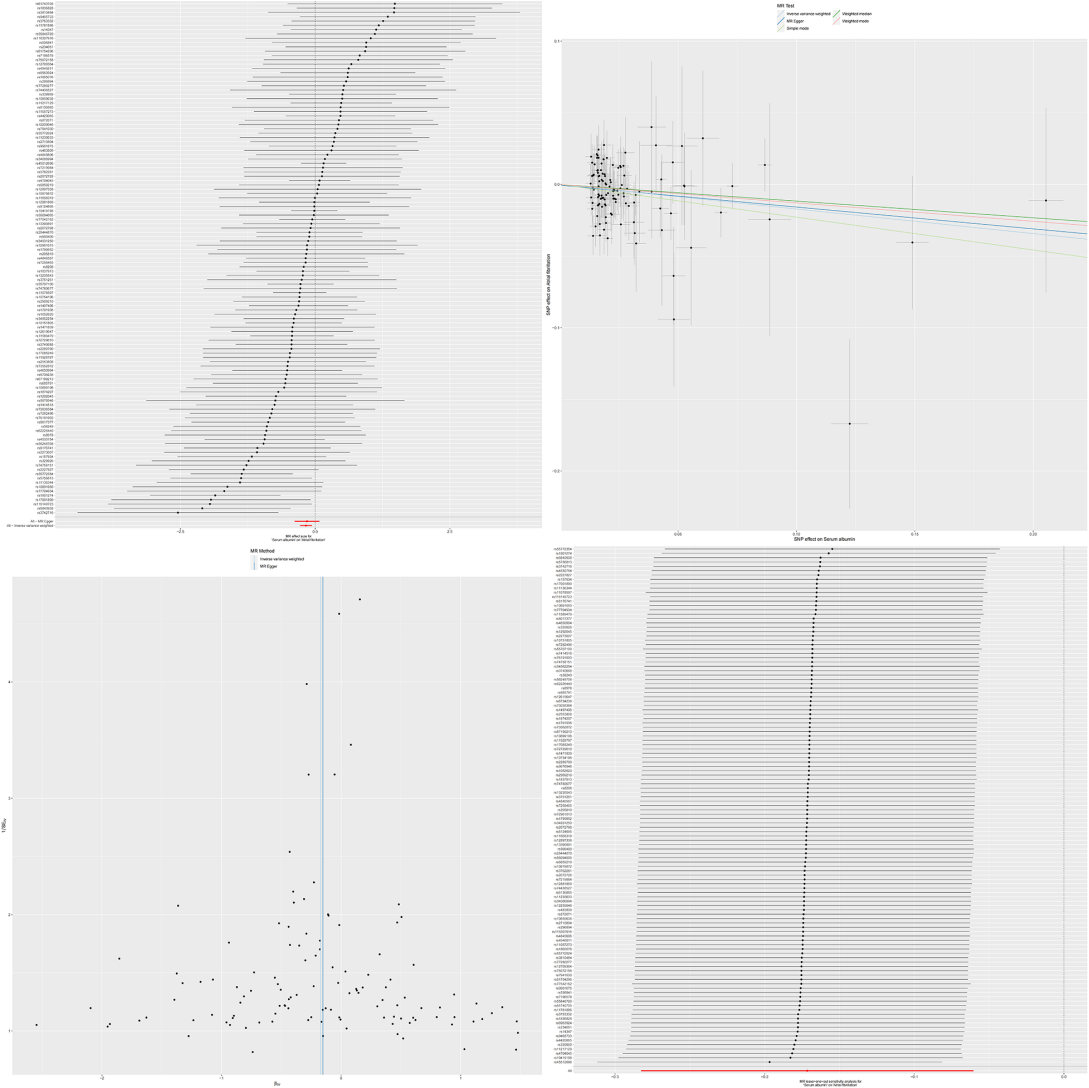
The effect value of instrumental variables. Leave-one-out sensitivity analysis of the MR effect of serum albumin and atrial fibrillation. The SNP effect on serum albumin.

To further verify the consistency and reliability of the instrumental variables, we used IVW and MR-Egger regression methods to detect heterogeneity among instrumental variables. The results of both methods indicate that there is no significant heterogeneity among instrumental variables. In addition, we also confirmed the absence of horizontal multi-effect interference in instrumental variables using the MR-PRESSO test. To further verify the stability of the MR results, we conducted a sensitivity analysis using the leave-one-out method. By removing SNPs one by one and comparing the causal effects of the remaining SNPs with the MR analysis results of all SNPs, we found that the MR results remained highly stable. This indicates that the causal relationship between serum albumin and reduced risk of atrial fibrillation is not dominated by a single or small number of instrumental variables, but is based on comprehensive data analysis and reliable conclusions (Figure 1). The results of MR analysis are shown in Supplementary Table S2.

### 3.2 Causal relationship between serum metabolite levels and atrial fibrillation

We systematically explored the potential causal relationship between 486 serum metabolites and atrial fibrillation. To ensure the accuracy and reliability of the results, we set a screening criterion of *P* value less than 1e-05 to screen out SNPs significantly associated with each metabolite. At the same time, the condition of F value greater than 10 eliminated weak instrumental variables, ensuring the validity of the analysis.

**Figure 2:**
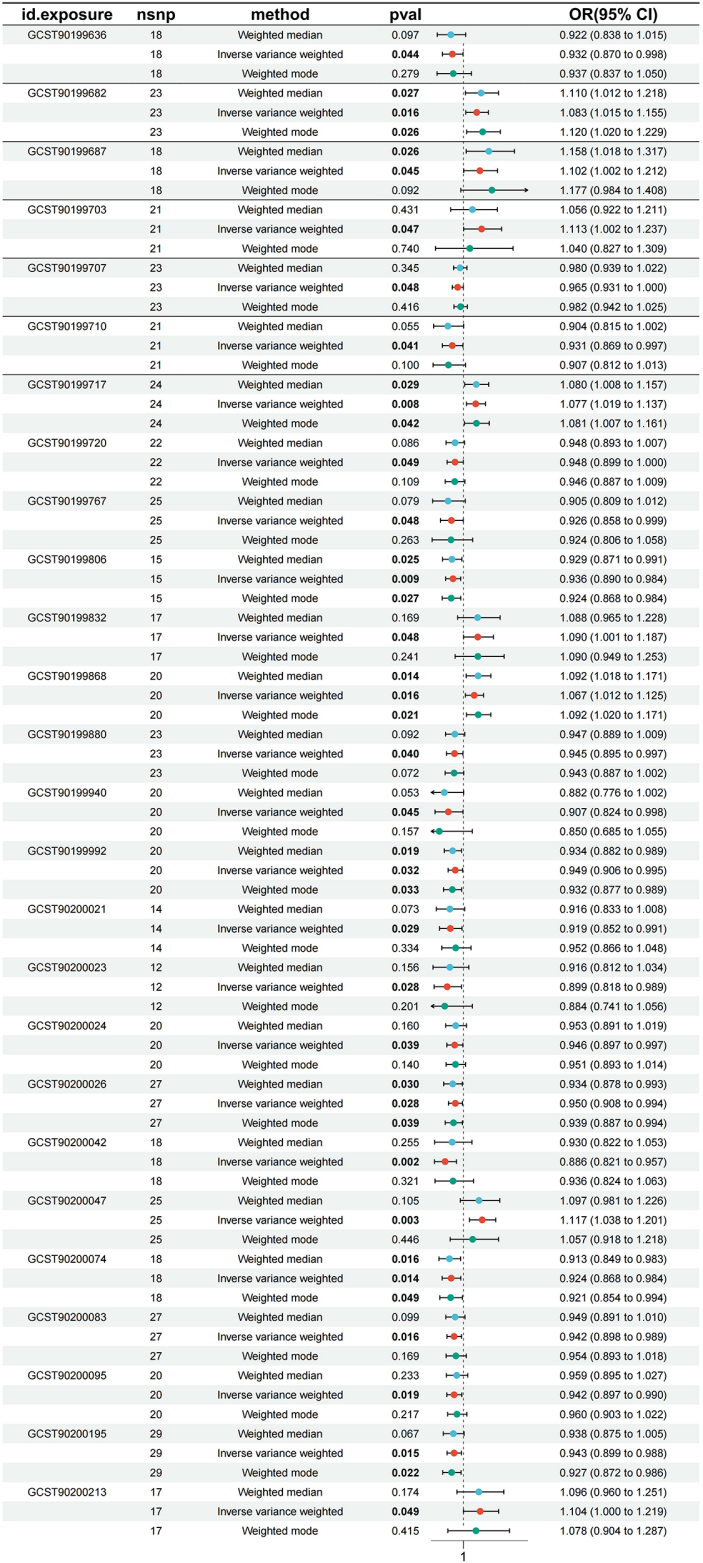
Forest plot of serum metabolites with atrial fibrillation.

In the process of in-depth analysis, we used five different analytical methods and required that the OR direction obtained by these methods must be consistent to enhance the confidence of the results. After a series of MR analyses, we found that a total of 26 serum metabolites had significant causal effects on atrial fibrillation. Specifically, kynurenine levels, pro-hydroxy-pro levels, docosatrienoate (22:3n3) levels, octanoylcarnitine (c8) levels, 5,6-dihydrouridine levels, tetradecanedioate (C14-DC) levels, cis-4-decenoylcarnitine (C10:1) levels, guaiacol sulfate levels, octadecanedioylcarnitine (C18-DC) levels, N-acetylglucosamine/n-acetylgalactosamine levels, trans 3,4-methyleneheptanoate levels, taurodeoxycholic acid 3-sulfate levels, glycodeoxycholate 3-sulfate levels, oleate/vaccenate (18:1) levels, 1-linoleoyl-GPG (18:2) levels, 1-(1-enyl-palmitoyl)-2-palmitoyl-GPC (P-16:0/16:0) levels, 1-linoleoyl-2-linolenoyl-GPC (18:2/18:3) levels had significant negative correlation with atrial fibrillation, which means they may help reduce the risk of atrial fibrillation. Homocitrulline levels, threonate levels, gamma-glutamylvaline levels, 1-methyl-4-imidazoleacetate levels, dimethylarginine (sdma + adma) levels, N-acetyl-3-methylhistidine levels, sphingomyelin (d18:1/17:0, d17:1/18:0, d19:1/16:0) levels and 2-naphthol sulfate levels showed significant positive correlation with atrial fibrillation and may be potential risk factors for atrial fibrillation. To verify the robustness of this result, we conducted sensitivity analysis on these 26 serum metabolites. The sensitivity analysis results support our conclusion that the results are stable. Figure 2 shows us a forest plot of serum metabolites with significant causal relationships with atrial fibrillation. The results of MR analysis are presented in Supplementary Table S3.

### 3.3 Causal relationship between serum albumin level and related serum metabolites

We further analyzed the exposure factor of serum albumin and selected 26 serum metabolites previously identified as having genetic causal relationships with atrial fibrillation as outcome variables for in-depth analysis. To ensure the rigor of the results, we set strict statistical significance thresholds (*P* < 5e-08) and instrumental variable screening criteria (*F* value > 10) to eliminate potential weak instrumental variables. At the same time, we used five different Mendelian randomization analysis methods, and required that the direction of the ORs obtained by these methods be consistent to enhance the confidence of the results.

When conducting mediation analysis with Mendelian randomization, we pay special attention to the independence of the genetic pathways between exposure factors and mediating variables, as well as between mediating variables and outcome variables. After careful screening, we confirmed that there are no identical SNPs in these two pathways that can be used as instrumental variables, thus satisfying the necessary conditions for conducting mediation analysis.

Through analysis using the IVW method, we revealed significant causal relationships between serum albumin and two specific serum metabolites. Specifically, there was a positive causal relationship between serum albumin and Docosatrienoate (22:3n3) levels (OR = 1.117, 95% CI: 1.001–1.247, *p* = 0.046), and similarly, a significant positive causal relationship was also observed between serum albumin and Oleate/vaccenate (18:1) levels (OR = 1.155, 95% CI: 1.051–1.271, *p* = 0.002). The results of MR analysis are shown in Supplementary Tables S4, S5.

**Figure 3:**
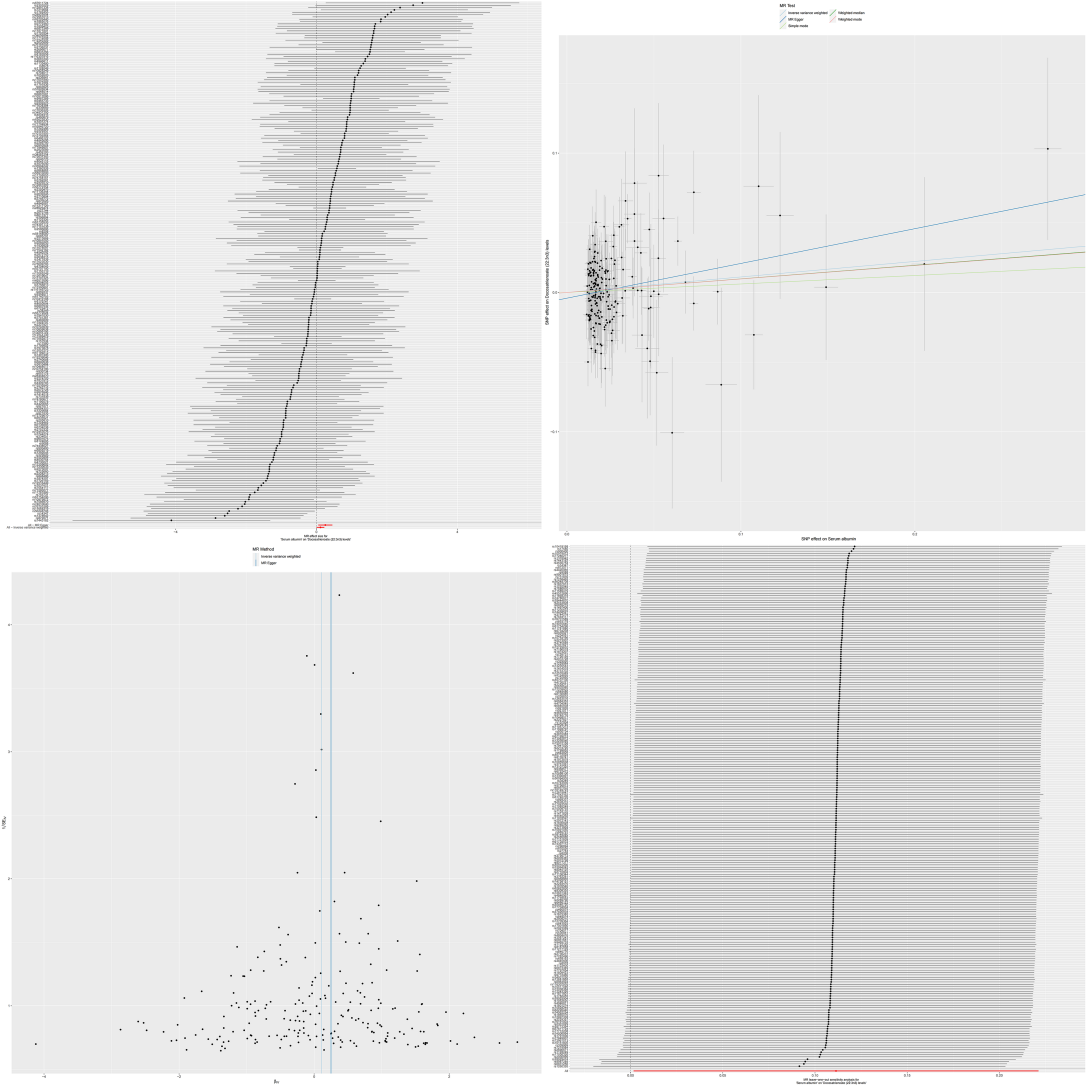
The MR effect size for serum albumin and docosatrienoate (22:3n3) levels. The effect value of instrumental variable. Leave-one-out sensitivity analysis of the MR effect of serum albumin and docosatrienoate (22:3n3) levels. The SNP effect on serum albumin.

**Figure 4:**
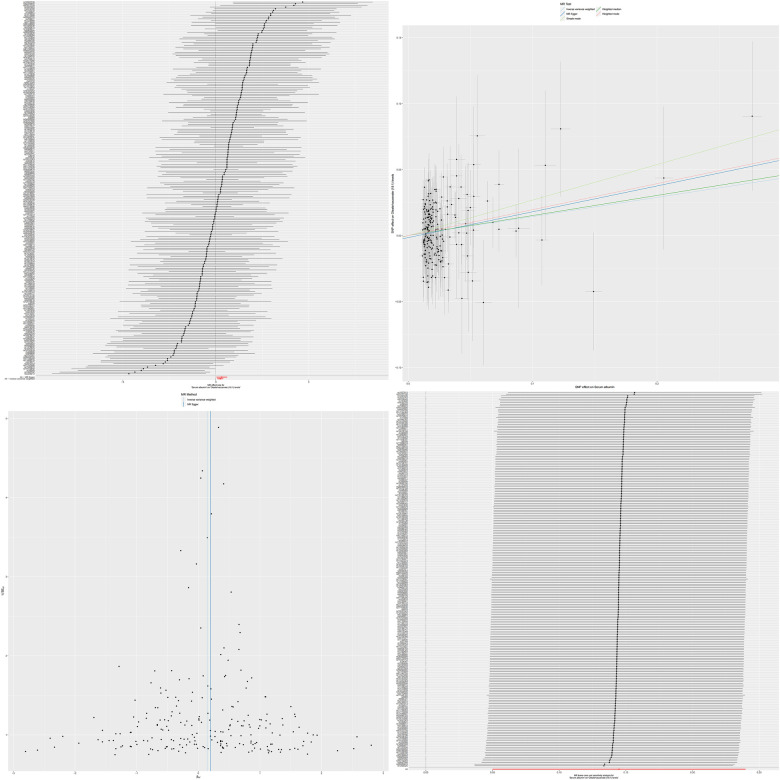
The MR effect size for serum albumin and oleate/vaccenate (18:1) levels. The effect value of instrumental variable. Leave-one-out sensitivity analysis of the MR effect of serum albumin and Oleate/vaccenate (18:1) levels. The SNP effect on Serum Albumin.

Combining our previous findings on the causal relationship between serum albumin levels and atrial fibrillation and serum metabolites and atrial fibrillation, we have reason to speculate that these two serum metabolites may play an important mediating role between serum albumin and atrial fibrillation (Figures 3, 4). The results of MR analysis are shown in Supplementary Tables S6, S7.

### 3.4 The mediating role of serum metabolites in the causal relationship between serum albumin and atrial fibrillation

**Figure 5:**
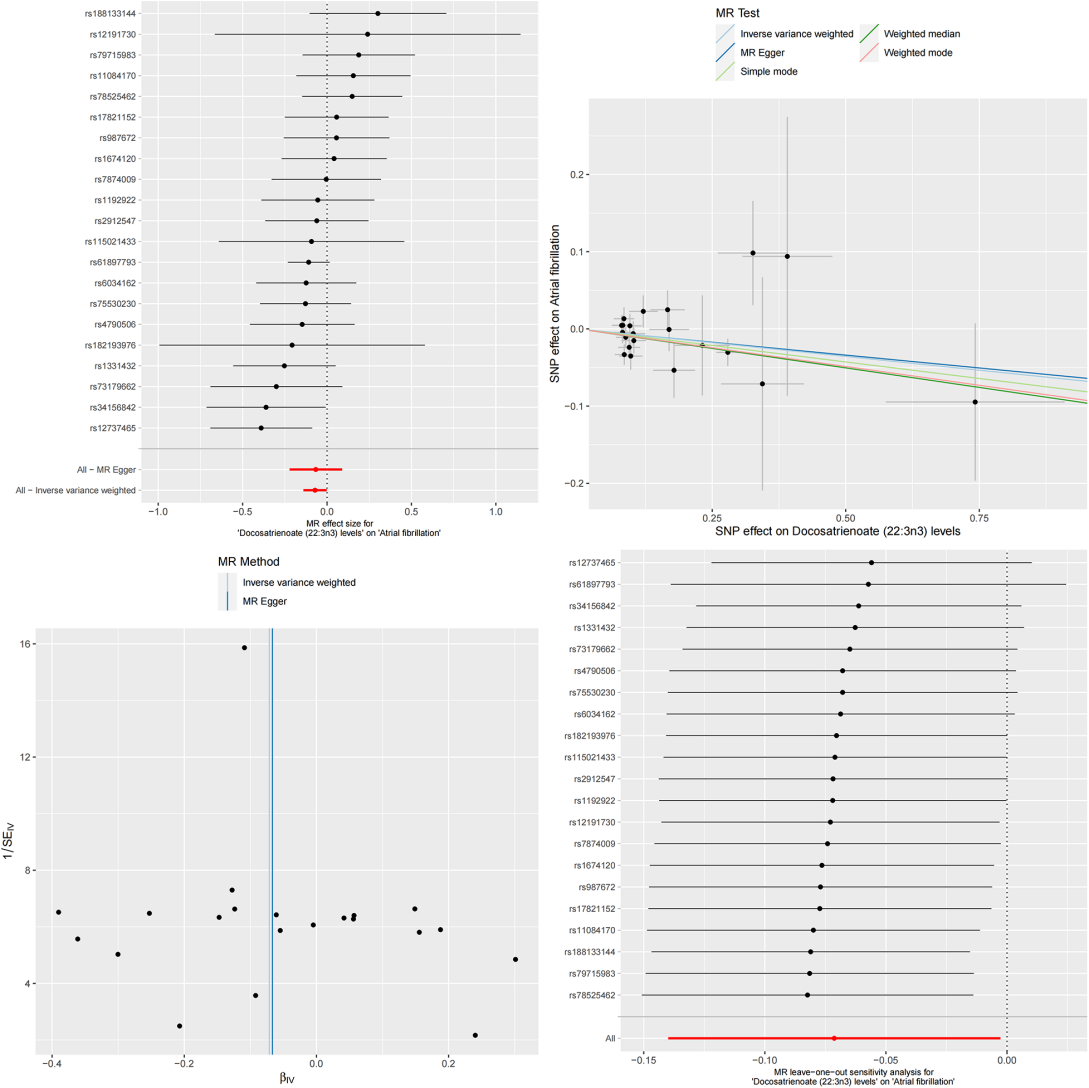
The MR effect size for docosatrienoate (22:3n3) levels and atrial fibrillation. The effect value of instrumental variable. Leave-one-out sensitivity analysis of the MR effect of docosatrienoate (22:3n3) levels and atrial fibrillation. The SNP effect on docosatrienoate (22:3n3) levels.

**Figure 6:**
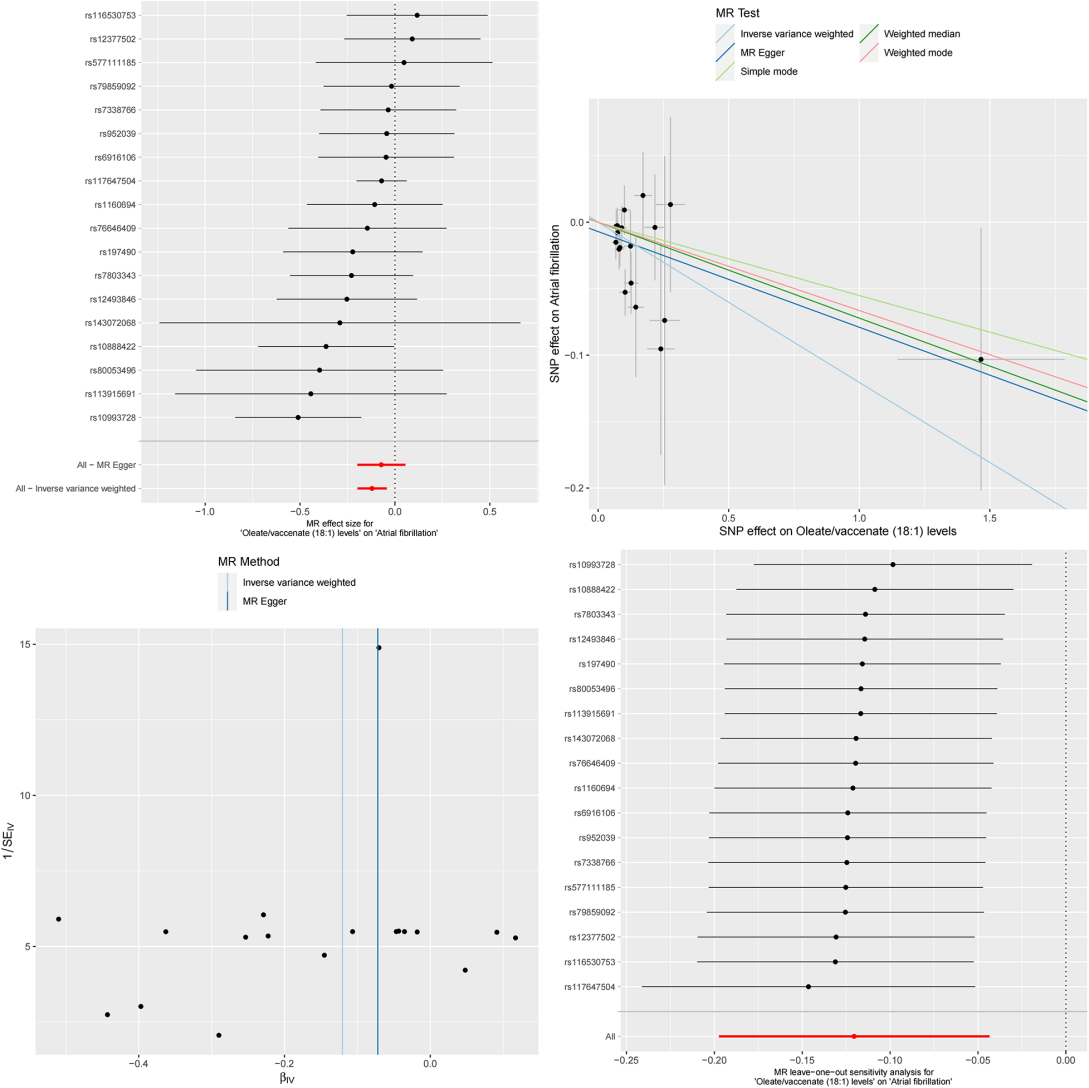
The MR effect size for oleate/vaccenate (18:1) levels and atrial fibrillation. The effect value of instrumental variable. Leave-one-out sensitivity analysis of the MR effect of Oleate/vaccenate (18:1) levels and atrial fibrillation. The SNP effect on Oleate/vaccenate (18:1) levels.

After a series of rigorous MR analyses, we successfully constructed a causal relationship chain based on the relationship between serum albumin and atrial fibrillation, mediated by the relationship between serum albumin and specific serum metabolites, and the relationship between these serum metabolites and atrial fibrillation. This series of causal links deeply reveals the key mediating role of these serum metabolites in the relationship between serum albumin and atrial fibrillation (Figures 5, 6).

To more accurately quantify this mediating effect, we conducted detailed calculations on these two specific serum metabolites. The results showed that this mediating effect had significant statistical significance. Specifically, Docosatrienoate levels played a mediating role in the causal relationship between serum albumin and atrial fibrillation, with a magnitude of effect of *β* = −0.008, SE = 3.271e-05, and a 95% confidence interval ranging from −0.008 to −0.007. This mediating effect accounted for 4.63% of the overall causal relationship, with a confidence interval of 4.59%–4.67%. Similarly, the mediating effect of Oleate/vaccenate levels was *β* = −0.017, SE = 7.157e-05, with a 95% confidence interval ranging from −0.018 to −0.017. This mediating effect accounted for up to 10.17% of the overall causal relationship, with a confidence interval of 10.09%–10.25%.

### 3.5 Validation using the Bayesian-weighted Mendelian randomization method

Finally, we validated our analysis using the Bayesian-weighted Mendelian randomization method. The results showed a significant negative correlation between serum albumin and atrial fibrillation (Beta = −0.172, OR = 0.842, 95% CI: 0.751–0.943, *P* = 0.003). Subsequently, we also verified the mediating role of serum metabolites in the causal relationship between serum albumin and atrial fibrillation. The results indicated a positive causal relationship between serum albumin and Docosatrienoate (22:3n3) levels (Beta = 0.113, OR = 1.120, 95% CI: 1.000–1.253, *p* = 0.049), as well as a significant positive causal relationship between serum albumin and Oleate/vaccenate (18:1) levels (Beta = −0.147, OR = 1.159, 95% CI: 1.050–1.279, *p* = 0.003). However, unfortunately, when verifying the causal relationship between the two metabolite levels, Docosatrienoate (22:3n3) and Oleate/vaccenate (18:1), with atrial fibrillation, we had to acknowledge that the Bayesian-weighted Mendelian randomization method did not successfully validate the causal relationship between Docosatrienoate (22:3n3) levels and atrial fibrillation (Beta = −0.072, OR = 0.931, 95% CI: 0.866–1.001, *p* = 0.053). Although the Beta value was less than 0 and the OR value was less than 1, indicating a negative causal relationship between Docosatrienoate (22:3n3) levels and atrial fibrillation, the *P*-value did not appear to be significant. On the other hand, we have successfully verified the causal relationship between Oleate/vaccenate (18:1) levels and atrial fibrillation (Beta = −0.154, OR = 0.857, 95% CI: 0.778–0.945, *p* = 0.001), which means that elevated Oleate/vaccenate (18:1) levels will reduce the risk of atrial fibrillation.

## 4 Discussion

Atrial fibrillation is a frequent arrhythmic phenomenon characterized by rapid and chaotic pulsations in the atria, which subsequently interfere with the normal rhythm of the ventricles. This condition significantly impairs the overall function of the heart, not only causing serious impact on patients' quality of life, but also potentially inducing other potential health problems (21). In the general population, the incidence of atrial fibrillation is approximately 2%, but in the elderly population aged 80 years and above, this proportion rapidly rises to 10%–12%. Various factors, such as myocardial fibrosis, inflammatory processes, oxidative stress, and genetic and behavioral factors, are involved in the development of atrial fibrillation to varying degrees, collectively weaving an intricate pathophysiological network (22). Albumin, a protein with a molecular weight of 69 kDa consisting of 585 amino acids, is one of the most abundant circulating proteins in the blood and accounts for more than half of the total serum volume (23). As a “transporter” of blood, albumin can not only bind to and transport a variety of endogenous and exogenous substances, but also serves as a core element in maintaining blood osmotic balance, which is crucial for ensuring the stable operation of the circulatory system. In addition, albumin also exhibits its multifaceted biological activities, including antioxidant, anti-inflammatory, and anti-platelet aggregation effects (24). In healthy adult populations, serum albumin concentration usually remains within a relatively stable range between 3.5 g/dl and 5 g/dl. It is worth noting that women's serum albumin levels tend to be slightly lower than those of men and show a gradual decreasing trend with age.

Previously, no study has used MR analysis to clarify the causal relationship between serum albumin level and the risk of atrial fibrillation. Our study is the first to fully utilize publicly available GWAS datasets for in-depth MR analysis, aiming to reveal the direct causal link between serum albumin level and atrial fibrillation. In addition, we further explored the rich genetic data of serum metabolites to explore potential mediating factors between the two. Our findings showed that a decrease in serum albumin level significantly increases the risk of atrial fibrillation, which is consistent with previous observational studies. This important finding provides new perspectives and conclusive evidence for a more comprehensive understanding of the pathogenesis of atrial fibrillation.

Although the potential mechanism of serum albumin reducing the risk of atrial fibrillation has not been conclusive, we can explore it from multiple perspectives. Firstly, the strong antioxidant capacity of albumin is a factor that cannot be ignored. The occurrence of atrial fibrillation is closely related to the increase in oxidative stress levels in the atrium. The excessive production of reactive oxygen species (ROS) is directly related to structural changes and electrophysiological remodeling of the heart, which not only plays a role in triggering atrial fibrillation, but also plays an important role in maintaining this arrhythmic state (25). Albumin contains a free thiol group, accounting for 80% of free thiols in plasma, which can selectively scavenge ROS, making albumin an important antioxidant and regarded as the most important antioxidant in whole blood (26, 27). In addition, serum albumin can carry nitric oxide and bilirubin in plasma, providing additional protection to the heart muscle and preventing oxidative stress (28).

Serum albumin may play another important role in preventing the risk of atrial fibrillation, which is inseparable from its inhibitory effect on inflammation and blocking the process of atherosclerosis. Inflammation exacerbates the electrophysiological and structural changes of the atrium through various pathways, thereby promoting the occurrence and maintenance of atrial fibrillation (29). Atherosclerosis will change the structure and function of gap junction proteins, increase the return of electrical signals and enhance the excitability of atrial tissue, further promoting the occurrence and development of atrial fibrillation (30). Endothelial dysfunction, as one of the early manifestations of atherosclerosis, is closely related to the adhesion and migration of leukocytes on the vascular endothelium, and is one of the important ways to trigger inflammation and atherosclerosis. Serum albumin can selectively intervene in this process, reduce adhesion between monocytes and endothelial cells, and thus alleviate inflammatory response (31). In addition, albumin can regulate the production of nitric oxide to improve endothelial function and alleviate persistent myocardial inflammation. This is achieved by reducing the messenger RNA and protein expression of myocardial nitric oxide synthase II (32). The decrease in serum albumin levels may affect the integrity of the endothelial layer of the heart, and supplementing with albumin can help maintain this important barrier and further reduce inflammatory responses (33). During the formation of atherosclerosis, the overactivity of platelets will release a variety of proinflammatory and growth factors, which will aggravate the formation of vasculitis and atherosclerosis. It is interesting that albumin can inhibit the ability of histone induced platelet aggregation activity, which is directly related to its concentration in the blood (34). In addition, albumin also has significant anticoagulant properties, which helps prevent changes in cardiac structure and electrophysiology caused by atherosclerosis, thus maintaining the stability of circulating blood flow and normal function of the heart (35).

Docosatrienoate is a polyunsaturated fatty acid (PUFA) belonging to the omega-3 family, which plays an important role in the composition of cell membrane phospholipids. The level of docosatrienoate is affected by multiple factors, including diet, age, gender, health status, and genetics. It decreases with age or the progression of chronic diseases (36). Omega-3 PUFA has been shown to affect cardiovascular health by regulating gene expression and transcription, promoting the production of anti-inflammatory lipids, regulating fat oxidation processes and free radical release, and inhibiting ectopic electrical activity (37–39). Specifically, it can directly act on ion channels in cardiomyocytes, stabilizing myocardial electrical activity by inhibiting the flow of specific ions (Na+, Ca2+, K+, etc.) and prolonging the relative refractory period of the atrium (40, 41). In addition, it can further optimize the function of ion channels by adjusting the fluidity of cardiomyocyte membranes, thereby enhancing the electrophysiological stability of myocardial tissue (42–44). Omega-3 PUFA can also effectively alleviate inflammation in cardiac tissue, reduce oxidative stress levels, and alleviate calcium overload in cardiomyocytes, which is attributed to its ability to inhibit the production and activity of pro-inflammatory cytokines while regulating nuclear factor activity (45–47). In addition, it has a optimizing effect on autonomic nervous function, balancing the activity of sympathetic and vagal nerves and improving endothelial function (45, 46). At the level of mitochondria, it can regulate mitochondrial calcium homeostasis, regulate mitochondrial gene expression, enhance mitochondrial respiration, and control ROS production, thereby protecting the heart from potential risks of mitochondrial damage and cell apoptosis in a comprehensive manner (48, 49). Finally, it is worth mentioning that omega-3 PUFA can precisely regulate the gene expression pattern of the heart and reduce the occurrence of arrhythmia (50, 51).

As a monounsaturated fatty acid (MUFA), oleate and its precursor oleic acid (OA) play an important role in the human circulatory system. They not only participate in fat metabolism as energy sources, but also constitute important components of cell membranes (52). Research has confirmed that OA can significantly reduce various cardiovascular risks (53). Its unique antioxidant properties are particularly noteworthy, as it can directly regulate the synthesis and activity of antioxidant enzymes, thereby helping to improve endothelial function (54). OA prevents the conversion of vasodilator molecule oxide nitric acid to harmful peroxynitrite under oxidative stress environment (55). OA can also further exert its antioxidant stress effects by reducing mediators associated with oxidative stress (56, 57). On the other hand, OA can regulate cholesterol transport and absorption, inhibit inflammatory factors, and thereby alleviate inflammation in the inner wall of blood vessels (58). In the immune system, OA also plays an important regulatory role. It can alter the fluidity of immune cell membranes (59), thereby affecting membrane-mediated signaling (60). In addition, OA can regulate the activity of immune cells by regulating multiple cytoplasmic signaling pathways such as MAPK and PI3 K/Akt (61). In neutrophils, OA can reduce their migration ability (62). In macrophages, it can inhibit the production of pro-inflammatory factors and promote the secretion of anti-inflammatory factors (63–65). OA can also affect the physiological processes of T cells and regulate the function of Treg cells (66–68). OA can also regulate the expression of various genes to further affect the function and activity of immune cells (69, 70).

Vaccinate, as a unique conjugated base of vaccenic acid(VA), is generated through the deprotonation process of carboxylic acid groups. It is worth mentioning that VA is a geometric isomer of OA, which also has various beneficial characteristics for cardiovascular health (71). Through research on various animal models, it has been found that VA can significantly improve the risk factors of cardiovascular disease. This positive effect may be attributed to its ability to reduce plasma cholesterol, regulate blood lipid levels, and optimize the ratio of high and low density lipoprotein (72–74). In addition, VA also demonstrated potential in reducing pro-inflammatory markers, successfully reducing IL-2 and TNF in rat disease models α The level of (75, 76). In addition, VA can regulate the production of arachidonic acid, which is a key bioactive substance, and has an important impact on regulating lipid metabolism, immune response, vascular function, platelet aggregation and other downstream metabolic pathways involved in atherosclerosis (77).

When exploring the relationship between serum albumin and serum metabolites, it is not difficult to discern the crucial role of albumin. Docosatrienoate, Oleate, and Vaccenate are all types of fatty acids. Our MR analysis revealed a significant positive correlation between serum albumin levels and these three fatty acids. However, it is inaccurate to say that serum albumin elevates fatty acid levels because serum albumin itself does not increase the synthesis or production of fatty acids. Instead, serum albumin's role in regulating fatty acids is primarily reflected in its binding, thereby exerting transport, homeostatic maintenance, and protective effects (78, 79).

Serum albumin in the human body is a vital plasma protein that serves as the main carrier for fatty acids. It possesses multiple binding sites for fatty acids, which exhibit varying affinities, including two high-affinity, five medium-affinity, and over 20 low-affinity binding sites (80, 81). As a result, serum albumin can tightly bind to multiple fatty acid moieties with varying degrees of affinity. Structurally, albumin is composed of a complex polypeptide chain that folds intricately to form several globular units. Interestingly, the strong-affinity fatty acid binding sites may be located precisely in the clefts between these globular units, providing an ideal binding environment for fatty acids (82). When fatty acids are present in their anionic form, they can bind tightly to albumin. This tight binding is primarily attributed to the non-polar interactions established between the fatty acid hydrocarbon chains and the uncharged amino acid side chains on albumin (83). It is worth noting that these binding sites exhibit a certain degree of flexibility, allowing their conformation to adjust flexibly to accommodate the binding needs of different fatty acids. This characteristic ensures that albumin can effectively bind to various fatty acids, playing a critical role in transport and regulation within the human body. This has been further confirmed by studies showing that when albumin is knocked out in mice, the concentration of free fatty acids in their plasma significantly decreases (84). This finding underscores the importance of albumin in maintaining fatty acid homeostasis and transport.

The binding of plasma albumin to fatty acids plays a crucial role in the human body. Firstly, the binding of albumin to fatty acids stabilizes their presence in the bloodstream, preventing their rapid degradation and ensuring their effective transport from the liver to other tissues and organs for utilization. Secondly, albumin actively participates in regulating fatty acid metabolism by binding to them, helping maintain a steady-state concentration of fatty acids in the plasma. Through precise binding and release of fatty acids, albumin regulates their distribution, utilization, and excretion in the body, supporting normal physiological functions of organs. Additionally, the binding action of albumin provides protection to fatty acids, shielding them from damage caused by oxidation and other harmful factors, thereby maintaining their biological activity. It is worth mentioning that serum albumin itself possesses significant antioxidant properties, further providing a protective barrier for fatty acids and reducing the risk of oxidative damage. In summary, the binding of plasma albumin to fatty acids not only ensures their stable transport and metabolism but also provides comprehensive protection for their safety and activity in the body.

More and more studies have revealed that there is a significant negative correlation between serum albumin levels and cardiovascular diseases, especially coronary atherosclerotic heart disease, heart failure and atrial fibrillation. Even after comprehensive consideration and correction of multiple risk factors, this association remains stable and evident (85, 86). It is worth mentioning that serum albumin, as a prognostic assessment tool, provides information independent of conventional prognostic markers and therefore has unique value (87). Especially in patients with atrial fibrillation who undergo ablation treatment, serum albumin levels can predict the risk of postoperative recurrence, further highlighting its important role in the pathological process of atrial fibrillation (88). A recent study has provided strong support for our findings, analyzing major electronic health record reports from 80 healthcare organizations in the United States between 2000 and 2023. Through this large-scale retrospective analysis of a joint research network, researchers have discovered that patients with low albumin levels face a significantly increased risk of cardiovascular disease. This increased risk is independent of factors such as age, gender, comorbidities, and other causes of hypoalbuminemia. Of particular concern is the strong association between low albumin levels measured during the acute phase and an increased risk of atrial fibrillation in clinical conditions characterized by increased oxidative stress, such as ischemic stroke. Specifically, a decrease in albumin levels is associated with an 11% increase in the risk of atrial fibrillation within 30 days of its occurrence (89).

In view of this, this study aims to deeply analyze the causal relationship between serum albumin and atrial fibrillation. We adopted the MR analysis method, which is a unique genetic perspective that can examine genetic variations closely related to risk factors (90). Through this approach, we hope to more accurately identify which risk factors directly contribute to the occurrence of atrial fibrillation.

Our study has the following advantages. Firstly, compared to traditional observational studies, MR analysis is more rigorous in exploring causal relationships and can effectively avoid the interference of reverse causality and confounding factors. Secondly, to ensure the accuracy and reliability of the study, we deliberately selected a large sample size, further enhancing the statistical power and minimizing the potential impact of insufficient data or uncertainties on the research results. Finally, we employed Bayesian Mendelian randomization for validation, enhancing the robustness and comprehensiveness of the findings, which aids in our more holistic understanding of the relationship between exposure factors and outcome variables.

Although we have made efforts to overcome various limitations in our research, we still need to face up to their existing limitations. When analyzing the causal relationship between serum albumin and atrial fibrillation, although we have tried our best to control other potential interfering factors, we still cannot completely rule out the possibility that SNPs related to serum albumin may affect atrial fibrillation through other indirect pathways. This potential link between this polymorphism and other traits may cause confounding effects, which poses a certain challenge to our causal inference. On the other hand,since we used summary statistics from GWAS only and not individual genotype data, the estimated causal effect may be biased due to various factors such as the use of single variants, genotype imputation errors, and heterogeneity of genetic effects across different populations. Even when F statistics >10 is used, the weak instrument variable problem still exists. Third one is, Although we have conducted MR analysis using large-scale GWAS data, more massive and comprehensive prospective cohort studies are needed in the future to further enhance the reliability of our research conclusions and gradually expand their scope of application.

## 5 Conclusion

In summary, our study strongly demonstrates the causal relationship between serum albumin and reduced risk of atrial fibrillation through genetic methods, and reveals the key mediating role of two serum metabolites in this relationship. These findings not only provide a new perspective for understanding the role of serum albumin in atrial fibrillation, but also provide innovative ideas and directions for the prevention and treatment of cardiovascular diseases such as atrial fibrillation.

## Data Availability

The original contributions presented in the study are included in the article/**Supplementary Material**, further inquiries can be directed to the corresponding authors.
